# Assessment of policy makers' individual and organizational capacity to acquire, assess, adapt and apply research evidence for maternal and child health policy making in Nigeria: a cross-sectional quantitative survey

**DOI:** 10.4314/ahs.v17i3.12

**Published:** 2017-09

**Authors:** Chigozie Jesse Uneke, Issiaka Sombie, Namoudou Keita, Virgil Lokossou, Ermel Johnson, Pierre Ongolo-Zogo, Henry Chukwuemeka Uro-Chukwu

**Affiliations:** 1 African Institute for Health Policy & Health Systems, Ebonyi State University, PMB 053 Abakaliki Nigeria; 2 Organisation Ouest Africaine de la Santé, 175, avenue Ouezzin Coulibaly, 01 BP 153 Bobo-Dioulasso 01, Burkina Faso; 3 Hopital Central Yaounde, CDBPH Lawrence VERGNE Building 2nd Floor, Avenue Henry Dunant Messa Yaoundé, Cameroon

**Keywords:** Policy maker, research, evidence, capacity, Nigeria

## Abstract

**Background:**

Throughout the world, there is increasing awareness and acknowledgement of the value of research evidence in the development of effective health policy and in quality health care practice and administration. Among the major challenges associated with the lack of uptake of research evidence into policy and practice in Nigeria is the capacity constraints of policymakers to use research evidence in policy making.

**Objective:**

To assess the capacity of maternal and child health policy makers to acquire, access, adapt and apply available research evidence.

**Methods:**

This cross-sectional quantitative survey was conducted at a national maternal, newborn and child health (MNCH) stakeholders' engagement event. An evidence to policy self-assessment questionnaire was used to assess the capacity of forty MNCH policy makers to acquire, assess, adapt and apply research evidence for policy making.

**Results:**

Low mean ratings were observed ranging from 2.68–3.53 on a scale of 5 for knowledge about initiating/conducting research and capacity to assess authenticity, validity, reliability, relevance and applicability of research evidence and for organizational capacity for promoting and using of research for policy making.

**Conclusion:**

There is need to institute policy makers' capacity development programmes to improve evidence-informed policymaking.

## Introduction/background information

In Nigeria, as in other parts of the world, the policymaking process is very well recognized as a complex one. Nonetheless, there is increasing awareness and acknowledgement in the country of the value of research evidence not only in the development of effective health policy but also in quality health care practice and administration^1^,^2^. Among the areas of health care where effective policy informed by research evidence is highly needed in Nigeria is maternal, newborn and child health (MNCH). Due to the weak health systems, maternal and child health outcome in Nigeria is poor with more than 1 million newborn, infant, and child deaths and more than 50,000 maternal deaths every year^3^–^5^.

There are many potential challenges related to research use in MNCH policy making and these challenges also affect other aspects of evidence-informed health policymaking process. Wilson and colleagues^6^, cited a number of such barriers that have been consistently identified from some studies across sectors. These barriers include: the complexity of research evidence, organizational barriers, lack of available time, poor access to current literature, lack of timely research, lack of experience and skills for critical appraisal, unsupportive culture for research, lack of actionable messages in research reports, and limited resources for implementation^7^–^11^. In addition to these, some previous reports have noted that the use of research evidence is but one factor influencing all stages of what is in practice described as ‘messy and political’ policy cycle^12^,^13^

The major challenges and barriers to the uptake of research evidence into policy and practice in low and middle income countries (LMICs) including Nigeria are associated with the capacity constraints of policy makers to acquire, access, adapt and apply available research evidence in policy making^2^,^14^,^15^. According to Dawad and Veenstra^16^, without adequate capacity, in knowledge translation/management and health policy research, policy makers will not have the capacity to access and synthesize sound information on which to base decisions and the potential for shared learning will be lost. Furthermore, Green and Bennett^17^, noted that knowledge and skill constraints associated with accessing evidence from various sources and competency in making use of the evidence appropriately are among the most important capacity needs of policy makers.

It is important to state that this capacity constraint regarding acquisition, assessment, adapting and applying research evidence in policy making is not only limited to the individual level but also at organizational level^2^,^14^. There is therefore an urgent need for the implementation of evidence-to-policy capacity enhancement mechanism that will target the improvement of both individual and organizational competencies.

However, in order to design an effective capacity enhancement strategy for evidence-to-policy process, there is need to assess the existing individual and organizational capacity to acquire, access, adapt and apply available research evidence in policy making. According to Deans and Ademokun^15^, before launching into capacity strengthening initiatives it is of paramount importance to understand what the existing capacities of policy makers are and which need to be developed. This position is supported by the United Nations Development Programme^18^ which states that capacity assessments are an essential step to developing rigorous and practical capacity development initiatives.

This study was aimed at identifying the capacity gaps at individual and organizational levels regarding MNCH evidence informed policy making in Nigeria from which lessons can be drawn for other LMICs. The study was designed to obtain data from national MNCH stakeholders including tacit knowledge and knowledge relating to evidence transfer. This was to serve as a first step towards effort to build national MNCH policy makers' capacity on issues related to knowledge transfer and use of evidence in MNCH policy making.

## Methods

The study design was a cross-sectional survey undertaken during a one-day national MNCH stakeholders' engagement event convened under the auspices of the West African Health Organization (WAHO) and the Federal Ministry of Health (FMOH) Nigeria, in October 2015 in Abuja, Nigeria. A cross-sectional quantitative survey was used because of the need to capture information based on data gathered for a specific point in time (ie., at the stakeholders' engagement event). The study design was also used to generate findings and outcomes that can be analysed in order to develop more in-depth research on policy makers' capacity constraints regarding evidence-informed policy making.

Among the objectives of the meeting was to assess the capacity of national policy makers and their organizations to acquire, assess, adapt and apply research evidence for MNCH policy making in Nigeria. Participants were officers of senior cadre drawn from the Federal Ministry of Health (FMOH) Abuja and its associated ministries, departments and agencies; others included directors from selected State Ministry of Health (SMOH); executive officers from development partner organizations (DPOs), civil society organizations (CSOs) and non-governmental organizations (NGOs). As part of the inclusion criterial, only individuals from the FMOH, SMOH, DPOs and CSOs/NGOs who were involved in MNCH programmes and policy making/implementation were invited.

A questionnaire was administered to participants after the completion of an informed consent form. The questionnaire and the informed consent form were approved by the University Research Ethics Committee of Ebonyi State University Nigeria (the institution of the principal author). The questionnaire was designed to assess participants' knowledge, capacity and organizational process of generation, synthesis and utilization of research evidence in policy making regarding MNCH. The questionnaire used for this assessment were modification of the self-assessment tool produced by the Canadian Health Services Research Foundation (CHSRF) available at the following websites:

- http://www.whatworksforchildren.org.uk/docs/tools/SelfAssessmtWEB.pdf
- http://www.cfhi-fcass.ca/sf-docs/default-source/documents/SAT-Self-Assessment-Tool.pdf

We chose the CHSRF self-assessment tool as the basis from which we developed our data collection tool because a number of reports have demonstrated that it can help projects evaluate their capacity to use research evidence in the design and delivery of services^19^–^22^.

The questionnaire contained core questions (hints), which assessed the following:

- acquisition of research evidence;
- assessing the validity, quality and applicability of research evidence;
- adapting the format of the researcher results to provide information useful to decision makers;
- application of evidence in decision making.

## Analysis of the questionnaire

The data collected via the questionnaires was analyzed using the methods developed at McMaster University Canada by Johnson and Lavis^23^ The main parameter measured was participants' perceptions of their own knowledge/understanding. The analysis is based on mean rating (MNR), median rating (MDR) and range. For instance, the figures represent Likert rating scale of 1–5 points, where 1 point = grossly inadequate; 2 points = inadequate; 3 points = fairly adequate; and 4 points = adequate; and 5 points=very adequate.

The range was recorded as the range of values represented by lowest number chosen from the response scale and the highest (e.g., 2–5). The mean was calculated as the numbers chosen from the response scale divided by the total number of responses to the question. While the median was determined by arranging the values chosen from the response scale in ascending order. In terms of analysis, values ranging from 1.00–3.49 points were considered low, whereas values ranging from 3.50–5.00 points considered high.

## Results

### Profile and official designation attributes of participants

A total of 40 participants signed the informed consent form and completed the questionnaire (Table 1). A total of 16 (40.0%) respondents were males, and most of the respondents (63.9%) were more than 44 years old. Majority of the respondents (45%) were from the Federal Ministry of health and its associated ministries, departments and agencies. Most of the respondents (59%) were either directors or chairpersons in their organizations, have either spent <3years (37.5%) or 3–5years (45%) in their present designation.

**Table 1:** Profile and official designation attributes of participants who completed the questionnaire at the stakeholders' engagement event.

Parameter assessed	Frequency (%)
**Gender**	
Male	16(40.0)
Female	24(60.0)
Total	40
**Age Category**	
25–34years	4(11.1)
35–44years	9(25.0)
>44years	23(63.9)
Total	36
**Type of organization**	
FMOH/MDAs	18(45.0)
SMOH	10(25.0)
NGOs/CSOs	5(12.5)
DPs	3(7.5)
Others	4(10.0)
Total	40
**Designation**	
Director	23(59.0)
Manager/Head of department	5(12.8)
Programme/project officer	11(28.2)
Total	39
**Duration in designation**	
<3years	15(37.5)
3–5years	18(45.0)
6–10years	5(12.5)
>10years	2(5.0)
Total	40

FMOH/MDAs = Federal ministry of health and and its associated ministries, departments and agencies; SMOH=State ministry of health; NGOs/CSOs=non-governmental organizations/civil society organizations; DPs=Development partners

### Acquisition of research evidence relevant to MNCH

The outcome of the assessment of acquisition of research evidence relevant to maternal, newborn & child health among the stakeholders' participants is presented in Table 2. The mean ratings (MNRs) of the participants' knowledge about initiating/conducting research, the capacity of their organization to initiate research and source for research evidence were low ranging from 3.21–3.41 on the scale of 5. The MNR of the level of research incentives available in participants' organizations was very low at 2.68 on the scale of 5 (Table 2).

**Table 2:** Outcome of assessment of capacity for acquisition of research evidence relevant to MNCH policy making among participants at stakeholders' engagement event in Nigeria.

(a). Individual research skill		*Ratings scale of 1–5		Number of responses
Parameter	Hints	Mean	Median	
(i). Adequacy of knowledge about initiating/conducting research	Identification of research problems; construction of research questions; designing of research methodology; writing of research proposals/ protocols; analysis & interpretation of research results; writing of research reports.	**3.41**	**4**	**39**
(ii). Ability to access and use existing research evidence	Journals, internet & library assess; non-journal reports eg. newspapers, textbooks, reports from national & international agencies, databases, websites; works from researchers & peers.	**3.64**	**4**	**36**
(b). **Institutional/Organizational incentive for research**				
(i). Organizational capacity to initiate research	Existence of research programs, departments, officers & facilities; any reputation on specialized areas of research; research outputs; regularity of research activities.	**3.39**	**4**	**38**
(ii). Organizational capacity to source for research evidence	Existence of databases; relationship with research institutions; collaboration with researchers & experts; commissioning of research projects.	**3.21**	**4**	**38**
(iii). Organizational level of research incentives	Availability of library, internet facilities; availability of research grants; award of honours/ promotions; in-service training; stipends, bonuses & per-diem for research works; provision of research facilities; sponsorship to conferences/workshops; institutional subscription of research materials (periodicals eg. journals), databases, websites.	**2.68**	**3**	**37**

* Ratings of response on a scale of 1 (grossly inadequate) to 5 (very adequate) MNCH= maternal, newborn & child health

### Assessing the validity, quality & applicability of research evidence relevant to MNCH

The outcome of assessing the validity, quality & applicability of research evidence relevant to maternal, newborn & child health among the stakeholders' participants is presented in Table 3. With reference to individual research skill, the mean ratings (MNRs) of the participants' capacity to assess the authenticity, validity, reliability & high quality of research evidence and capacity to assess the relevance and applicability of research evidence were low and ranged from 3.29–3.43 on the scale of 5. In terms of the organizational incentive for research, the mean ratings (MNRs) of the participants' organizations having incentives to encourage the application of research evidence in general and necessary incentives for assessment of the validity, quality and applicability of research evidence were very low ranging from 2.77–2.78 (Table 3).

**Table 3:** Outcome of assessment of capacity for assessing the validity, quality & applicability of research evidence relevant to MNCH policymaking among the participants at stakeholders' engagement event in Nigeria

(a). Individual research skill		*Ratings scale of 1–5		Number of responses
Parameter	Hints	Mean	Median	
(i). Capacity to assess the authenticity, validity, reliability & high quality of research	1. The skill to evaluate & appropriate the quality of research methodology	**3.37**	**3**	**37**
	2. The skill to evaluate the reliability of specific research evidence and to compare research methods and results.	**3.29**	**3**	**35**
(ii). Capacity to assess the relevance and applicability of research evidence	1. The skill to identify relevant similarities and differences between research evidence.	**3.43**	**3**	**37**
	2. The skill to evaluate the differences in the research evidences in the context of your organization.	**3.42**	**3**	**36**
(b). **Institutional/Organizational incentive for** **research**				
(i). Organizational incentives for assessment of the validity, quality and applicability of research evidence	Use of consultants; application of computer software, statistical package; well-equipped laboratory; existence of quality control units; promotion of ethical standards/practices.	**2.77**	**3**	**39**
(ii). Organizational incentives to encourage the application of research evidence	Availability of research evidence implementation committee; availability of administrative process for accepting/implementing research evidence.	**2.88**	**3**	**40**

* Ratings of response on a scale of 1 (grossly inadequate) to 5 (very adequate) MNCH+ maternal, newborn & child health

### Adapting the format of the research results to provide information useful to decision makers relevant to MNCH

The outcome of adapting the format of the research results to provide information useful to decision makers relevant to maternal, newborn & child health among the participants is presented in Table 4. With reference to individual research skill, the mean ratings (MNRs) of the participants' ability to summarize research results in a user-friendly way and ability to present results of research to decision makers ranged from 3.28–3.66 on the scale of 5. In terms of the organizational incentive for research, the mean ratings (MNRs) of the participants' organizations having incentives to encourage the provision of research evidence to decision makers was 3.0 on a scale of 5 (Table 4).

**Table 4:** Outcome of assessment of capacity for adapting the format of the research results to provide information useful to decision makers relevant to MNCH policy making among the participants at stakeholders' engagement event in Nigeria.

(a). Individual research skill		*Ratings scale of 1–5		Number of responses
Parameter	Hints	Mean	Median	
(i). Ability to summarize research results in a user-friendly way	1. Present research results concisely in accessible language	**3.56**	**3**	**34**
	2. Synthesize in one document relevant research as well as information and analysis from other sources.	**3.28**	**3**	**36**
	3. Link the research results to key issues and provide recommendations.	**3.53**	**4**	**35**
(ii). Ability to present results of research to decision makers	Use of charts, tables, graphs, pictogram, bullet/power point presentations, etc.	**3.66**	**4**	**38**
(b). **Institutional/Organizational incentive for** **research**				
Organizational incentives to encourage the provision of research evidence to decision makers	Summarized and easy-to-use research evidence is routinely brought to the attention of relevant decision makers (such as through regular meetings or reports; or participation by researchers/analysts in management meetings to present/discuss evidence).	**3.00**	**3**	**36**

* Ratings of response on a scale of 1 (grossly inadequate) to 5 (very adequate) MNCH+ maternal, newborn & child health

### Application of evidence in decision making relevant to MNCH

The outcome of adapting the format of the research results to provide information useful to decision makers relevant to maternal, newborn & child health among the participants is presented in Table 5. In terms of the participants' organizations leading by example on how research use is valued in general and in maternal, newborn & child health specifically, the mean ratings (MNRs) were generally very low ranging from 2.85–3.18 on a scale of 5. The assessment of participants' organization's decision-making processes having a place for research indicated the mean ratings (MNRs) that are also generally very low ranging from 2.91–3.09 on a scale of 5 (Table 5).

**Table 5:** Outcome of assessment of capacity for application of evidence in decision making relevant to MNCH policymaking among the participants at stakeholders' engagement event in Nigeria

Institutional/Organizational incentive for research		*Ratings scale of 1–5		Number of responses
Parameter	Hints	Mean	Median	
(i). Organization leading by example on how research use is valued	1. Using research is a priority: our organization has committed sufficient people, time, training and budgets to access, appraise, adapt and apply research in making decisions	**3.18**	**3**	**39**
	2. Our organization's job descriptions and performance incentives include enough focus on activities which encourage using research.	**2.85**	**3**	**38**
	3. Both management and front-line staff support and participate in frequent forum where staff and invitees present and discuss research evidence related to the organization's main goals.	**2.95**	**3**	**38**
	4. Management has clearly communicated corporate strategy and priority areas for improvement, so that people creatingor monitoring research evidence know what is needed.	**2.94**	**3**	**35**
	5. Our organization has effective communication channels so that priorities, evidence and ideas are exchanged across divisions, as well as between management and front lines.	**2.94**	**3**	**34**
	6. Our corporate culture is to value and reward flexibility, change, and continuous quality improvement, and we provide adequate resources at all levels to support change.	**3.00**	**3**	**35**
(ii). Organization's decision making processes having a place for research	1. When we make major decisions, we usually allow enough time to identify researchable questions and create/ obtain, analyze and consider research results and other evidence.	**2.91**	**3**	**33**
	2. Our management team has enough expertise to evaluate the feasibility of each option, including potential impact across the organization as well as on its clients, partners and other stakeholders.	**3.09**	**3**	**35**
	3. When staff develop or identify high quality and relevant research, decision makers will usually give formal consideration to any resulting recommendations.	**3.00**	**3**	**34**
	4. Staff and appropriate stakeholders know when and how major decisions will be made, how and when they can contribute evidence and how that information will be used	**2.97**	**3**	**33**
	5. The staff who have provided evidence and analysis usually participate in the discussion before a decision is made and, when possible, so do relevant non-staff researchers	**3.06**	**3**	**34**
	6. When a decision is made, feedback to staff and appropriate stakeholders includes a rationale for the decision, and review of how the available evidence influenced the choices made	**3.00**	**3**	**35**

* Ratings of response on a scale of 1 (grossly inadequate) to 5 (very adequate) MNCH=maternal, newborn & child health

## Discussion

The outcome of the assessment of individual capacity to acquire, assess and adapt research evidence relevant to MNCH among the participants generally indicated low MNRs. However, there are areas where the MNRs were very low suggesting areas of greatest capacity need. In some previous studies, that made use of the CHSRF self-assessment tool, the areas of lowest ratings indicated areas of greatest capacity constraints^2^,^14^,^20^–^22^. From the present study, any mean rating below 3.5 is considered poor and indicates weak capacity. In terms of capacity to acquire evidence, the mean rating was as low as 3.41 regarding knowledge about initiating/conducting research, which included the ability to effectively identify research problems; construct research questions; design research methodology; write research proposals/protocols; analyze/interpret results and write research reports. In terms of individual skill to assess the authenticity, validity, reliability and high quality of research evidence regarding evaluating the reliability of specific research evidence and to compare research methods and results, the mean rating was low at 3.29. And in terms of the ability to adapt evidence regarding summarizing research results in a user-friendly way as well as synthesizing in one document relevant research as well as information and analysis from other sources the mean rating was low at 3.29.

These findings were not a surprise occurrence as they clearly confirmed earlier reports which indicated that many policy makers particularly the administrators lack research skill to acquire, assess and adapt evidence, which has led to a general lack of understanding about how evidence can be used properly^24^–^25^. In a recent systematic review by Humphries and co-workers^26^, it was observed from a number of studies reviewed that a deficit in the skills and experience of decision-makers in research literacy and research utilization, and a lack of formal management training were expressed as barriers to evidence-use in program management^6^,^7^.^27^,^28^. In an assessment of community capacity to acquire, assess, adapt, and apply research evidence relevant to HIV/AIDS, Wilson and colleagues^6^ noted that capacity was lowest for the domains related to: acquiring research; assessing the reliability, quality, relevance, and applicability of research evidence; and summarizing results in a user-friendly way. This is clearly an area where there is a gap and will require some attention in any capacity enhancement strategy for policy makers.

The mean ratings of the assessment of level of research incentives available in participants' organizations for acquiring, assessing, adapting and applying research evidence was generally very low. The worst outcome was reordered in terms of the organizational capacity for applying research evidence in which majority of the mean ratings ranged from 2.79–2.97. These findings clearly suggest that there is still a huge gap in terms of the organizational structure in place to support the use of research evidence in policy making in Nigeria. For instance, organizational incentive for research acquisition was as low as 2.68 regarding internet facilities; availability of research grants; in-service training, stipends, bonuses & per-diem for research works; provision of research facilities; sponsorship to conferences/workshops etc.

Similarly, organizational incentives for assessing the validity, quality and applicability of research evidence was as low as 2.77 especially concerning existence of quality control units; use of consultants/experts; application of computer software, statistical package; and promotion of ethical standards/practices. Also in terms of the organizational capacity to adapt evidence regarding research evidence implementation committee and availability of administrative process for accepting/implementing research evidence the mean rating was very low at 2.78.

These findings clearly indicate that there is a very poor research enabling environment in the participants' organizations. Similar organizational capacity constraints were identified in a number of previous studies as serious barriers to evidence informed policy making process. Wilson and co-workers^6^, in their study, found that overall organizational capacity to acquire, assess, adapt, and apply research evidence was low. They also noted that overall, the community based organizations studied were not only limited in incentive, and resources for assessing the quality and reliability of research, but they also have limited arrangements with external experts to help them. The factors related to organizational structure and process identified in a previous study, included lack of research structure, research, planning or decision-support positions; and under-resourcing described as an inability to allocate resources to research or evidence-related positions^28^.

Among the key organizational factors incapacitating evidence to policy process identified by some other studies was a lack creation of a learning culture and institution of good management - that promote better decision-making^29^–^32^. Bowen and colleagues^28^ also identified the lack of information technology (IT) resources, including lack of databases or staff to support them and ensure data quality as key organizational constraint. In the further analysis of issues related to data constraints, four main components have been identified including: lack of data (availability and timeliness); lack of systems and resources for tracking, organizing and retrieving data; data overload; and lack of access to library resources, or capacity to conduct literature searches^28^. The greater capacity gap at organizational than at individual level is not a reporting bias. In fact, it would have been surprising if the result turned out otherwise. The improved awareness of the need for policy to be informed by the best available evidence has led many policy makers to initiate processes to improve their individual capacity for research. Unfortunately, there is yet to be a commensurate effort to improve the process at organizational level. Therefore, this study reflects the true picture of what is obtainable at organizational level in Nigeria policy making sector.

This development is worrisome and poses a critical challenge to the evidence-informed policy-making process regarding MNCH in Nigeria, because inadequate organizational capacity and commitment towards evidence-informed policy making can incapacitate even the highly knowledgeable and skillful policy maker^1^. It is very obvious that the lack of enabling research environment and incentives in a policy making organization will further demoralize a policy maker in such organization who may want to make a deliberate effort to use evidence for policy making. It will therefore amount to a daunting task improving the poor maternal and child health outcomes in Nigeria with this high level of capacity constraints for evidence informed policymaking at individual and organizational levels. Nigeria is reportedly having more than 10% of all under-5 and maternal deaths world wide^3^,^4^,^33^. Strengthening the capacity of policymakers and their organizations for evidence informed policymaking holds great prospects for improved MNCH outcomes in Nigeria.

## Conclusion

Although the emphasis that policy makers/decision takers ought to be skilled enough to find, appraise evidence or even conduct research may be based on a futile premise. However, it is our opinion that this capacity constraint may be playing contributory to weak health policy making and implementation in Nigeria. Irrespective of the busy schedule of policy makers, if they have better research skill they may find the evidence-informed policy making process a lot easier. This study is intended to promote the awareness of need for capacity enhancement at national level and the employment of people with research capacities in policy making positions. The study has provided base line scientific information that will be vital to the development of capacity enhancement training programmes on evidence-to-policy process targeted at policy makers at all levels of operation and governance in Nigeria.

## Study limitation

One of the main limitations of this study was the rather relatively small study sample size of 40. A larger sample size may have provided better insights regarding the study outcome. However, our confidence and reliability of the study comes from the fact that we specifically targeted key national policy makers involved in MNCH programmes, and policy making/implementation. Individuals from the invited organizations who were not involved in MNCH were excluded from the study and that accounted for the limited number. Because there was substantial representation of the MNCH policymakers from the federal and states ministries of health, the findings can to a reasonable extent be generalised as far as FMOH and SMOH are concerned in Nigeria. We used the study to gain understanding at a small scale regarding the MNCH policy makers' capacity constraints on evidence-informed policy making in order to develop more in-depth research. A much more robust research methodology than a simple cross-sectional study and which involves expanded scope of MNCH stakeholders is required in future research on this important subject.
